# Introducing crucial protein panel of gastric adenocarcinoma disease 

**Published:** 2017

**Authors:** Mostafa Rezaei-Tavirani, Majid Rezaei-Tavirani, Vahid Mansouri, Seyed Mohammad Mahdavi, Reza Valizadeh, Mohammad Rostami-Nejad, Mohammad Reza Zali

**Affiliations:** 1 Proteomics Research Center, Shahid Beheshti University of Medical Sciences, Tehran, Iran; 2 Faculty of Medicine, Iran University of Medical Sciences, Tehran, Iran; 3 Physiotherapy Research Center, Shahid Beheshti University of Medical Sciences, Tehran, Iran; 4 Faculty of Medicine, Ilam University of Medical Sciences, Ilam, Iran; 5 Basic and Molecular Epidemiology of Gastrointestinal Disorders Research Center, Research Institute for Gastroenterology and Liver Diseases, Shahid Beheshti University of Medical Sciences, Tehran, Iran; 6 Gastroenterology and Liver Diseases Research Center, Research Institute for Gastroenterology and Liver Diseases, Shahid Beheshti University of Medical Sciences, Tehran, Iran

**Keywords:** Gastric adenocarcinoma, Protein-protein interaction network, Gene ontology, Hub-bottleneck nodes, Biomarker panel

## Abstract

**Aim:**

Since interactome analysis of diseases can provide candidate biomarker panel related to the diseases, in this research, protein-protein interaction (PPI) network analysis is used to introduce the involved crucial proteins in Gastric adenocarcinoma (GA).

**Background:**

Gastric adenocarcinoma (GA) is the most common type of stomach cancer. There is no efficient diagnostic molecular method for GA.

**Method:**

Applying Cytoscape software 3.4.0 and String Database, the PPI network was constructed for 200 genes. Based on centrality parameters, the critical nodes were screened. Gene ontology of the key proteins for pathway analysis and molecular function processing were done and the highlighted pathways and activities were discussed.

**Results:**

Among 200 initial genes, 141 genes were included in a main connected network. Seven crucial proteins, including tumor protein p53, epidermal growth factor receptor, albumin, v-erb-b2 erythroblastic leukemia viral oncogene homolog 2, neuro/glioblastoma derived oncogene homolog (avian), v-akt murine thymoma viral oncogene homolog 1, v-src sarcoma (Schmidt-Ruppin A-2) viral oncogene homolog (avian) and catenin (cadherin-associated protein), beta 1, 88kDa, and Myogenic differentiation 1, were introduced as key nodes of the network. These identified proteins are mostly involved in pathways and activities related to cancer.

**Conclusion:**

In conclusion, the finding is corresponding to the significant roles of these introduced proteins in GA disease. This protein panel may be a useful probe in the management of GA.

## Introduction

Mortality of gastric cancer as one of the main lethal kind of cancer, approximately were unchanged over 30 years. Several types of cancer are diagnosed in stomach that gastric adenocarcinoma (GA) is the most common ones (1). Genetics, nutrition and *Helicobacter pylori* are introduced as risk factors of GA (2, 3). Endoscopy and biopsy is efficient tools in GA diagnosis. This aggressive tool is used in the advanced stages of the disease (4). There are different studies regarding the role of various genes relative to GA (5, 6). The high-throughput studies showed that the vast range of gene expression alterations is happening in various stages of GA (7, 8). However a numerous involved genes are introduced, but there is no common molecular method for diagnosis of GA (9). Application of PPI network analysis in medicine has attracted the attention of scientists (10). Interactome analysis can provide a useful information about molecular map of diseases (11). In this method, many proteins or genes related to a disease are collected and matched to construct a network, including linked nodes by edges (the link is called edge). Each protein (as a node) in the network interacts with the certain proteins depend on the reciprocal affinity between them (12). The several important topological indices for a network are centrality parameters. Degree, betweenness and closeness are three well known centrally parameters that are used frequently for PPI network analysis. The numbers of edges that connect directly to a node are known as degree (K) and a node with high degree value is called a hub node. The betweenness centrality of a node (for example node n) is calculated in the following steps: fist, all possible paired nodes in the network (except the node n) are determined. Second, the ratio of number of shortest paths between a paired nodes that pass through node n relative to the number of all shortest paths between this paired nodes are determined. Third, the summation of all calculated ratios that its value (BC) is; 0≤BC≤1, and therefore called betweenness of node n. Two nodes of the network may be connected by multiple pathways; the path includes a minimum number of edges is called distance or shortest path (11). A node with high value of betweenness is called a bottleneck node (13). The node with high amounts of degree and also betweenness values is known as hub—bottleneck node (14). Closeness the other centrality parameter is defined as; inverse of the average value of the length of the shortest paths that pass through a node. As like as betweenness, the amounts of closeness centrality (CC) are in the range of 0-1 (11).

 There are numerous genes that their regulation depends on the incidence and advances of a disease (15-17). This relationship is discovered via classical research or high-throughput investigation (18-20). Therefore “Which one of them is a critical involved gene in the disease?” is a challenging question in medicine. One important screening method in this case is PPI network analysis (21). The genes rank based on their topological properties in the interactome unit. Therefore, an analysis of the vast range of the genes leads to a reduced and restricted suggested biomarker panel (22, 23). Gene ontology can be used to determine the involved molecular functions, biological processes, cellular components and biological pathways of the analyzed proteins (24). In this study, 200 related genes to gastric adenocarcinoma were provided from string database, corresponded PPI network constructed by Cytoscape software and the network was analyzed topologically. 

## Material and Methods

Different sources are available for providing related proteins to diseases. One of the important sources is Cytoscape 3.4. This common software is free and is compatible with different sources. Cytoscape and its applications are powerful tools to provide useful data and information for the mapping PPI network. One of the well-known interaction sources is a String Database (SD) (http://string-db.org/) (25, 26). Access to SD is possible through Cytoscape software. Three options of SD are protein, PubMed and disease queries. In this paper 200 related genes to gastric adenocarcinoma are retrieved from a disease query of SD. The corresponded PPI network was constructed and topological parameters were determined. The used cut off for interaction evidence was set at 0.5. Topological analysis provided information about degree, betweenness and closeness centralities. The disease score that shows the relation between the disease and the obtained protein was determined. The nodes with a high value of the degree (connections) are known as hub nodes. The 20 top nodes based on degree values are selected as hub genes. Betweenness centrality (BC) of a node refers to the amount of its exerted control on the other nodes. The nodes with high value of betweenness are called bottlenecks (27). These elements are crucial for the disease onset and progress (28). The hub nodes with high betweenness value are considered as hub-bottleneck nodes (27). In this research the cutoff for degree and betweenness are 60 and 0.03 respectively. Gene ontology analysis of the crucial nodes was done by the application of ClueGO. The ontology analysis was based on pathway analysis and molecular function (MF). The pathways that include at least 4 genes and the genes were at least 4% attributed in the pathway are selected as the relevant pathways. The pathways are grouped and the group was nominated by the name of the pathway that include most number of the genes. The terms that include at least 2 genes and the genes were at least 3% attributed in the term are selected as the involved MF. The MFs are grouped and the group was nominating by the name of the MF that include most number of the genes. In each cluster, similar enrichments were included (29). 

## Results

**Figure 1:**
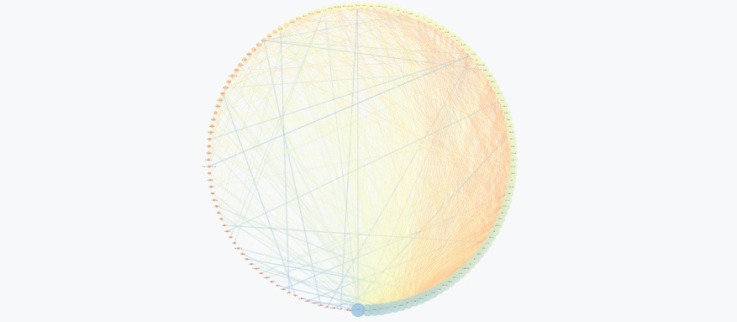
The main connected component of gastric adenocarcinoma PPI network. The network consists of 200 nodes, including 57 isolated nodes, one pair nodes and 141 connected nodes. The main connected component includes 141 nodes and 1508 edges. The nodes are arranged by degree value (as the nodes get bigger, the degree increases) and are layout via circular mode. From orange to blue color the degree values were increased. In the right-down position of the figure, the edge density is in max values. Similar to degree pattern, the edges’ colors are also arranges based on edge betweenness values

**Figure 2:**
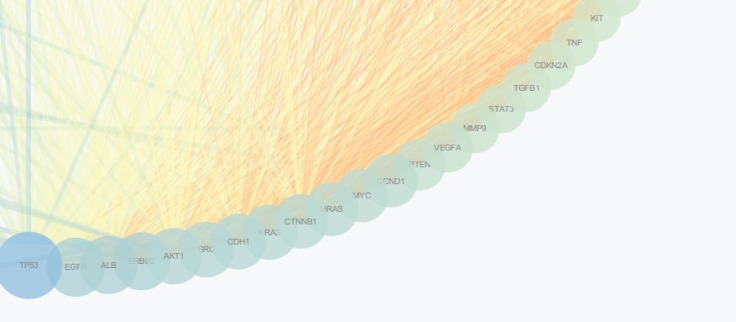
Schematic representation of 20 top nodes (based on degree) among 141 represented nodes in figure 1. TP53 is the first ranked node and KIT is the 20^th^. The other nodes are arranged between these mentioned proteins

**Figure 3:**
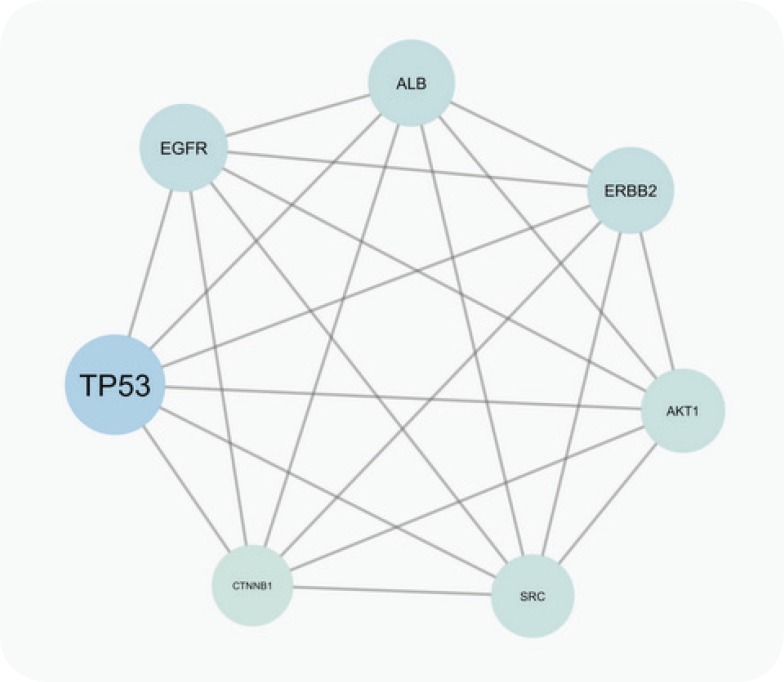
The main connected component of gastric adenocarcinoma PPI network. All nodes exception seven crucial nodes (the introduced nodes in the table 2) are removed from the network. There are 21 edges between the seven nodes (each node is connected to the six nodes).

**Figure 4:**
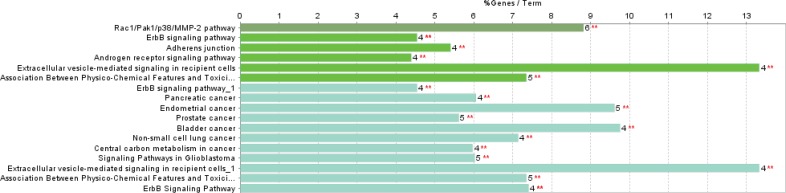
The results of pathway analysis for the seven key nodes by clueGO. The minimum percentage of attribution of the genes in the term and minimum numbers of the involved genes in same term are considered as 4% and four genes respectively. P values in maximum amount were less than 0.001. The terms with similar color are grouped in an individual group

**Figure 5:**
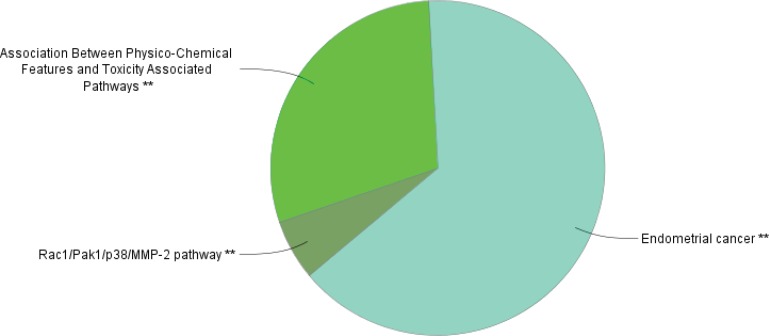
Schematic representation of percentage of attribution of the grouped terms (the introduced terms in figure 4). Each groups are nominated by the term that includes maximum genes (If there are two similar terms the considered term possess more percentage of attribution

**Figure 6:**
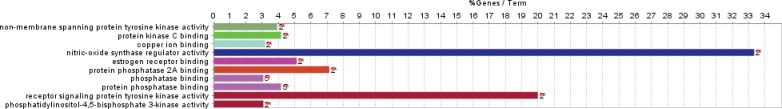
The results of molecular function analysis for the seven crucial nodes by clueGO. The minimum percentage of attribution of the genes in the MF and minimum numbers of the involved genes in the same MF are considered as 3% and two genes respectively. P values in maximum amount were less than 0.001. The MFs with similar color are grouped in an individual group

**Figure 7:**
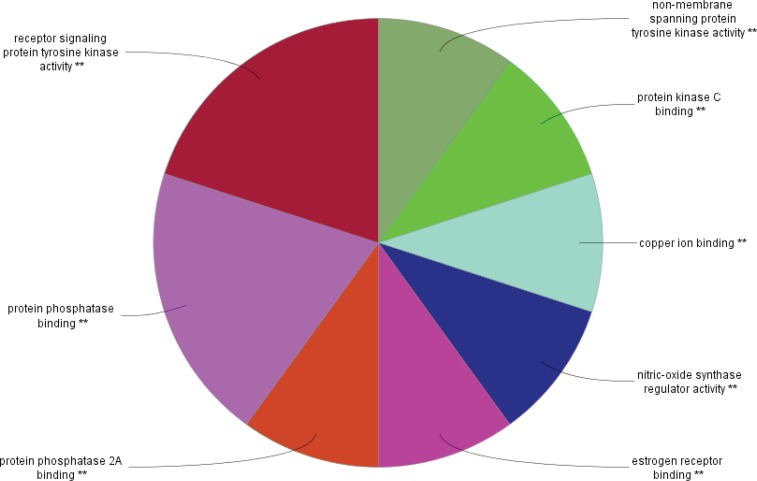
Schematic representation of percentage of attribution of the grouped MF (the introduced MF in figure 4). Each groups are nominated by the MF that includes maximum genes (If there are two similar MF the considered MF possess more percentage of attribution

The PPI network for gastric adenocarcinoma was constructed by 200 nodes from String databank. The network includes 57 isolated nodes, one paired nodes and a connected component of 141 edges. This component includes 141 nodes and 1508 edges (see figure 1). For better resolution 20 top nodes based on degree values are selected and the other nodes were deleted from the network (the nodes and the related edges are represented in figure 2). The name of the 20 nodes and their centrality parameters (degree, betweetness and closeness) and also disease scores are presented in table 1. To reduce the number of 20 hub nodes and to achieve to crucial genes, the nodes with degree less than 60 and betweenness under 0.03 were deleted. Seven key proteins were selected (hub-bottleneck proteins) and their characteristic parameters are shown in table 2. This panel including, tumor protein p53, epidermal growth factor receptor, albumin, v-erb-b2 erythroblastic leukemia viral oncogene homolog 2, neuro/glioblastoma derived oncogene homolog (avian), v-akt murine thymoma viral oncogene homolog 1, v-src sarcoma (Schmidt-Ruppin A-2) viral oncogene homolog (avian) and catenin (cadherin-associated protein), beta 1, 88kDa, and Myogenic differentiation 1. The nodes of the connected component (exception these seven nodes) were deleted and the reminded nodes were shown in figure 3. This subnetwork includes seven nodes and 21 edges. Since pathway analysis is a useful tool to determine the role of an individual protein, the pathway analysis of seven key proteins were done and the findings are represented in the figures 4 and 5. Molecular function analysis can provide useful information about the role of the studied proteins (24). In figures 6 and 7 the results of molecular analysis for the introduced seven hub-bottleneck proteins are shown. The minimum percentage of attribution of the genes in the term was 4%. At least presence of four genes in term was regarded. The P value of maximum amount was less than 0.001. The terms with similar color are grouped in an individual group. 

**Table 1:** Presentation of the selected 20 hub nodes for GA. The elements of the table are sorted by degree values and are corresponded to the represented nodes in figure 2. The amounts of betweenness centralities (BC), closeness centralities (CC) and disease scores are presented in the columns 4-7

**R**	**name**	**Description**	**Degree**	**BC**	**CC**	**disease score**
1	TP53	tumor protein p53	89	0.13	0.72	1.98
2	EGFR	epidermal growth factor receptor	71	0.05	0.65	1.50
3	ALB	Albumin	69	0.05	0.64	0.88
4	ERBB2	v-erb-b2 erythroblastic leukemia viral oncogene homolog 2, neuro/glioblastoma derived oncogene homolog (avian)	69	0.07	0.65	1.99
5	AKT1	v-akt murine thymoma viral oncogene homolog 1	65	0.05	0.63	1.23
6	SRC	v-src sarcoma (Schmidt-Ruppin A-2) viral oncogene homolog (avian)	64	0.06	0.61	1.25
7	CDH1	cadherin 1, type 1, E-cadherin (epithelial)	64	0.02	0.62	2.09
8	KRAS	v-Ki-ras2 Kirsten rat sarcoma viral oncogene homolog	63	0.02	0.62	0.86
9	CTNNB1	catenin (cadherin-associated protein), beta 1, 88kDa	61	0.03	0.63	1.69
10	HRAS	v-Ha-ras Harvey rat sarcoma viral oncogene homolog	60	0.02	0.61	0.96
11	CCND1	cyclin D1	59	0.04	0.61	1.29
12	MYC	v-myc myelocytomatosis viral oncogene homolog (avian)	59	0.04	0.61	1.49
13	PTEN	phosphatase and tensin homolog	55	0.02	0.58	0.97
14	VEGFA	vascular endothelial growth factor A	53	0.01	0.58	1.41
15	MMP9	matrix metallopeptidase 9 (gelatinase B, 92kDa gelatinase, 92kDa type IV collagenase)	50	0.02	0.58	1.21
16	STAT3	signal transducer and activator of transcription 3 (acute-phase response factor)	50	0.01	0.56	0.96
17	TGFB1	transforming growth factor, beta 1	49	0.01	0.56	0.99
18	CDKN2A	cyclin-dependent kinase inhibitor 2A	48	0.02	0.58	1.34
19	TNF	tumor necrosis factor	47	0.02	0.56	0.97
20	KIT	v-kit Hardy-Zuckerman 4 feline sarcoma viral oncogene homolog	46	0.01	0.55	0.91

**Table 2:** The seven determined hub-bottleneck nodes of the human gastric PPI network. The characterized nodes with degree value≥60 and betweenness centrality ≥ 0.03 are selected as hub-bottleneck nodes

**R**	**name**	**Description**	**Degree**	**BC**	**CC**	**disease score**
1	TP53	tumor protein p53	89	0.13	0.72	1.98
2	EGFR	epidermal growth factor receptor	71	0.05	0.65	1.50
3	ALB	Albumin	69	0.05	0.64	0.88
4	ERBB2	v-erb-b2 erythroblastic leukemia viral oncogene homolog 2, neuro/glioblastoma derived oncogene homolog (avian)	69	0.07	0.65	1.99
5	AKT1	v-akt murine thymoma viral oncogene homolog 1	65	0.05	0.63	1.23
6	SRC	v-src sarcoma (Schmidt-Ruppin A-2) viral oncogene homolog (avian)	64	0.06	0.61	1.25
7	CTNNB1	catenin (cadherin-associated protein), beta 1, 88kDa	61	0.03	0.63	1.69

## Discussion

Protein interaction mapping as a molecular and screening probe, attracted scientist’s attention and as a powerful analytical method is applied in a medical investigation (30). In network medicine topological, features of a specific disease are assessed for introducing the crucial involved genes or proteins in the disease. The information which introduce several essential proteins in terms of interactions that can be key proteins in disease onset and progression (31). These proteins can be considered as diagnostic or therapeutic biomarkers that by validation tests may be introduce for clinical approaches. The aim of this study was management and analysis of known and involved genes in GA disease for better understanding of molecular aspects of disease and screening of the genes. It was expected that a limited numbers of important genes be highlighted as a biomarker panel for GA disease. Consequently, as it was shown in figure 1 a network of all 200 top reported proteins for GA was constructed. Only 141 nodes participate in formation of network as a main connected component. There are 1508 edges that link the nodes of the network. The nodes of the network are sorted based on degree value. For better understanding the top 20 nodes (the nodes with higher values of degree) were selected and shown in the figure 2 and table 1. Tumor protein p53 and v-kit Hardy-Zuckerman 4 feline sarcoma viral oncogene homolog with degree values 89 and 46, are located in the up and down of table 1, respectively. This primary list of nodes was screened based on BC. The nodes with degree≥60 and BC≥0.03 selected as hub-bottleneck genes. Surprisingly, the six top nodes in table 1, (with the similar priority) and CTNNB1 as the 9^th^ node were remained after the screening (see table 2). The CC is also approximately follows the similar trend comparing with K and BC. It seems that these seven proteins are important in the network integrity. In figure 3, the main connected component (presented in figure 1) shows that all nodes are omitted except these seven nodes. All nodes are connected to the six neigbor nodes directly. There are 21 edges in this sub-network. As discussed, these nodes are densely connected. Since, for each disease, there are specific related pathways, it seems that pathway analysis for these seven proteins can provide essential evidences that confirm the crucial roles of these protein panel in GA. The involved pathways of the seven critical proteins are shown in figure 4. There are 17 pathways in three clusters that at least four proteins among seven introduced proteins are included in each pathway. Six proteins are attributed to the Rac1/Pak1/P38/MMp-2 pathway. The regulatory role of this pathway in angiogenesis in ovary cancer is well known (32). There are five proteins in the five pathways that mostly belong to various cancers. Minimum and maximum values of percentage attribution of these proteins in pathways are 4 and 14%, respectively. Based on figures 4 and 5, approximately all determined pathways are related to cancer. One of the biochemical features of diseases is regulatory changes of many enzyme or protein activities (33, 34). This alteration is related to expression changes of many involved genes. Molecular function analysis for a certain protein set is a useful tool to reveal the importance role of that protein in incidence and advances of the diseases (24). As shown in figures 6 and 7, molecular function analysis shows the crucial proteins are involved in 10 biochemical functions that categorized in eight clusters. Five proteins are related to the each phosphatase binding and protein phosphatase binding activities. Two proteins participate in the other activities. The most rate of attribution (33%) happened in nitric-oxide synthesis regulatory activity. There are three isoforms of nitric oxide synthases family, which are involved in cancer. This activity is reported in tumor cells of several histogenetic origins and is detected together the important aspects of cancer grade and development. The high level of this activity is associated with inhibition of tumor progression and reduced activity have is accompanied with tumor growth promotion (35).

In conclusion, there is a closed possible biomarker panel related to the gastric cancer. The pathway analysis and molecular function assessment are corresponding to the crucial role of these highlighted proteins. Investigation in the field can be a useful validation method for feasible application of the findings. 
